# The influence of socio-economic status and multimorbidity patterns on healthcare costs: a six-year follow-up under a universal healthcare system

**DOI:** 10.1186/1475-9276-12-69

**Published:** 2013-08-20

**Authors:** Raymond N Kuo, Mei-Shu Lai

**Affiliations:** 1 Institute of Health Policy and Management, College of Public Health, National Taiwan University, Taipei City, Taiwan; 2 Center of Comparative Effectiveness Research, National Center of Excellence for Clinical Trial and Research, National Taiwan University Hospital, Taipei City, Taiwan; 3 Institute of Epidemiology and Preventive Medicine, College of Public Health, National Taiwan University, Taipei City, Taiwan

## Abstract

**Introduction:**

Multimorbidity has been linked to elevated healthcare utilization and previous studies have found that socioeconomic status is an important factor associated with multimorbidity. Nonetheless, little is known regarding the impact of multimorbidity and socioeconomic status on healthcare costs and whether inequities in healthcare exist between socioeconomic classes within a universal healthcare system.

**Methods:**

This longitudinal study employed the claims database of the National Health Insurance of Taiwan (959 990 enrolees), adopting medication-based Rx-defined morbidity groups (Rx-MG) as a measurement of multimorbidity. Mixed linear models were used to estimate the effects of multimorbidity and socioeconomic characteristics on annual healthcare costs between 2005 and 2010.

**Results:**

The distribution of Rx-MGs and total costs presented statistically significant differences among gender, age groups, occupation, and income class (p < .001). Nearly 80% of the enrolees were classified as multimorbid and low income earners presented the highest prevalence of multimorbidity. After controlling for age and gender, increases in the number of Rx-MG assignments were associated with higher total healthcare costs. After controlling for the effects of Rx-MG assignment and demographic characteristics, physicians, paramedical personnel, and public servant were found to generate higher total costs than typical employees/self-employed enrolees, while low-income earners generated lower costs. High income levels were also found to be associated with lower total costs. It was also revealed that occupation and multimorbidity have a moderating effect on healthcare cost.

**Conclusions:**

Increases in the prevalence of multimorbidity are associated with higher health care costs. This study determined that instances of multimorbidity varied according to socioeconomic class; likewise there were inequities in healthcare utilization among individuals of various occupations and income levels, even when demographic characteristics and multimorbidity were controlled for. This highlights the importance of socioeconomic status with regard to healthcare utilization. These results indicate that socioeconomic factors should not be discounted when discussing the utilization of healthcare by patients with multimorbidity.

## Introduction

Multimorbidity has been defined two ways: the co-occurrence of two or more conditions in an individual within a specified period of time [1,2]; and the coexistence of multiple illnesses of different types [3]. Previous studies have reported the prevalence of multimorbidity ranging from 55 to 98% among older populations and approximately 20–30% when a comprehensive range of ages was included [4]. Clinical guidelines tend to focus on single morbidities rather than the coexistence of multiple conditions and traditional payment structures generally favor services that target single diseases or conditions. As a result, healthcare systems can face considerable challenges in the treatment of patients with multimorbidity and this issue has been attracting considerable attention, particularly in countries with an aging population.

Multimorbidity has been linked to elevated healthcare utilization [4-6]. New evidence suggests that increases in the use of resources cannot be attributed to an increase in chronic illness, but rather to the number of interventions required to deal with multiple health-related conditions [3,7]. Previous studies have shown that patients of lower socioeconomic status tend to use more health care services, primarily due to a higher prevalence of multimorbidity [8]. In addition, socioeconomic status has been shown to be an important factor associated with multimorbidity [4,9,10]. In a study by Schäfer et. al., income was associated with the number of chronic conditions [11]. Agborsangaya et. al. reported that the prevalence of multimorbidity was lower among those reporting higher incomes [12]. Nonetheless, very few studies addressing the issues of multimorbidity and healthcare utilization have included occupation as a factors in the measurement of socioeconomic status [2]. This study sought to fill this gap.

Although the relationship between multimorbidity and healthcare utilization has previously been explored, little is known regarding the impact of multimorbidity on healthcare costs within a universal healthcare system, such as that found in Taiwan. Taiwan launched its National Health Insurance (NHI) program in 1995. More than 98% of the population is currently enrolled in the NHI program [13], and over 90% of all healthcare facilities are contracted by the Bureau of National Health Insurance (BNHI) [14]. The NHI program provides universal access to healthcare and comprehensive benefits, including inpatient and ambulatory care, dental services, prescribed medications, nursing care, traditional Chinese medicine therapy, and preventive services. Co-payment for health services is also waived for low income earners. This raises the question of whether inequities (referring to differences which are unnecessary and avoidable and, in addition, are also considered unfair and unjust [15]) in healthcare exist between socioeconomic classes under a universal healthcare system.

This longitudinal study employed the claims database of the National Health Insurance of Taiwan using a mixed linear modeling approach to explore the influence of socio-economic status, (as measured by occupation and income level) and multimorbidity patterns on healthcare costs.

## Methods

### Data sources

This study employed the National Health Insurance Database of Taiwan 2005 (LHID2005) and obtained National Health Insurance (NHI) claims data for these individuals throughout the period from 2005 to 2010. This provided a population representative cohort that includes nearly one million of the 25.68 million NHI enrolees. No significant differences have been identified between the patients in the LHID2005 and the general population with regard to the distribution of gender, age, or average monthly income [13]. We used the LHID2005 to collect patient IDs, dates of ambulatory or inpatient care, annually total costs, and the medications prescribed for each case between January 1, 2005 and December 31, 2010. In addition to the above claims data, we employed the National Health Insurance Enrolment file within the NHID to identify the socioeconomic status of patients, including their occupation and level of income. The medical personnel registry (PER file) was also used to determine whether any of the patients was a physician or medical professional. For patients who were unemployed, data related to the insured spouse or relative was used.

All case IDs required for data linkage were encrypted prior to release. According to the privacy protection laws and regulations of Taiwan, the data used in this study possesses no unique patient identifier or any sensitive private information that could be traced back to individual patients.

### Sample selection criteria

This study included enrolees of the National Health Insurance, sampled from the LHID2005 database. Individual cases with discontinued enrolment in the NHI program for an entire year between 2005 and 2010 were excluded from the study, because they were deemed to have had less opportunity to access health care covered by the NHI. Thus, the costs of such cases would have been prone to under estimation. The final sample size for 2005 used in this study was 959 990 (96%).

### Measure of socioeconomic status

This study designated socioeconomic status according to occupation and income level. The categories used for occupations in this study included employers, public servants, physicians, paramedical personnel, farmers, fishermen, other employees/self-employed, and low income earners (as recognized by the government, regardless of work performed). According to NHI policy regarding co-payments, enrolees who are recognized as low income earners are excused from having to pay co-payments for inpatient or outpatient care. The monthly, salary-based income of patients (or insured) was categorized into four levels: above USD$3,850, USD$1,210 - $3,850, USD$594 - $1,210, below USD$594 (USD 1 = NTD 30). A binary variable was also used to identify patients who were an unemployed spouse or relative.

### Measure of multimorbidity

This study selected the medication-based Rx-defined morbidity groups (Rx-MGs) within the Johns Hopkins Adjusted Clinical Groups (ACG) system to measure multimorbidity [16]. The ACG system was developed to predict healthcare utilization and costs based on groupings of diagnoses [3,16-18] and medications [19]. Although diagnosis-based models were widely adopted, literature shows that prescription data are superior for the prediction of pharmaceutical-related costs [16,20] and total costs [21-23]. Compared to diagnosis data, prescription data is often more reliable, complete, and less of a gamble [24-26]. The Rx-MG algorithm assigns each instance of medication use into 1 of the 64 Rx-MGs, according to the following criteria: primary anatomico-physiological system, morbidity differentiation, expected duration, and severity [16,27]. An international mapping algorithm within the ACG system is used for Rx-MG assignment based on the WHO Anatomical Therapeutic Chemical (ATC) classification to classify medication-related data collected outside the U.S. [28]. Previous studies verified the effectiveness of medication-based Rx-MGs in predicting pharmaceutical costs and total medical costs in Taiwan [19]. Nine of the sixty-four Rx-MGs are used for minor, acute symptoms or diseases (e.g. acute and minor allergies) and were therefore excluded. Previous studies have demonstrated that the ACG/Rx-MGs outperforms other diagnosis-based morbidity measures in explaining variations in cost and predicting future healthcare utilization [29].

### Data analysis

This study sought to estimate the effect of multimorbidity and socioeconomic characteristics on annual healthcare costs. Because all cases were followed from 2005 to 2010 (with the exception of those who died during this period), we employed general linear mixed models with repeated measurements to account for the clustering of factors (measured one time annually). This approach is well suited to data of this nature, particularly with regard to the estimation of parameter of each covariate presented in models.

This study analyzed data using both annual total costs and non-pharmaceutical related costs comprising all claims to the National Health Insurance from outpatient visits, inpatient treatment, and community pharmacies. Dollar values are presented, as well as the *relative weight* (RW) provided by bivariate analysis, calculated as the cost of each category of cases divided by the mean population. Prescriptions in the LHID2005 include those dispensed at outpatient clinics, in-hospital pharmacies, and community pharmacies. Prescription codes from the claims data were first mapped to the WHO ATC codes, and then entered into the Johns Hopkins ACG system for Rx-MG assignment. Multimorbidity was measured by counting the number of Rx-MG assignment attributed to each enrolee each year. The control variables included age and gender.

This study fitted three alternative models for log-transformed total annual costs and log-transformed annual non-pharmaceutical costs. The first model controlled for age and gender then estimated effect of occupation and income on log-transformed costs. The second model also included multimorbidity (Rx-MGs classification) and the third model included the interaction between occupation and multimorbidity. All statistical operations were performed using SAS (version 9.2, SAS Institution Inc.).

## Results

A total of 959,990 NHI enrolees were followed from January 2005 to December 2010, resulting in 903,376 cases following exclusions due to death or withdrawal from the NHI program. Characteristics of the enrolees selected from the National Health Insurance Database are shown in Table 1. Enrolees exceeding 50 years of age accounted for more Rx-MG assignments (mean = 4.9) than younger people. Farmers and low income earners accounted for more Rx-MG assignments than those of other occupations (mean = 5.9 and 6.5, respectively). Physicians (and their relatives) accounted for fewer Rx-MG assignments; however, their annual total healthcare costs (RW: 1.03) and non-pharmaceutical costs (0.97) were higher than those of paramedical personnel (RW: 0.74 and 0.76), public servants (RW: 0.91 and 0.90), and other employees/self-employed (RW: 0.85 and 0.85). Low income earners had considerably higher costs than other enrolees, which may be attributed to the high prevalence of multimorbidity and the co-payment waiver. We also found that the distribution of Rx-MG counts and total costs presented statistically significant differences among gender, age groups, type of occupation, and income class (p < .001).

**Table 1:** Characteristics of the NIH enrolees included in this study, 2010

				**Count of Rx-MGs**		**Total cost**		**Non-pharmacy cost**	
		**n**	**%**	**Mean**	**(S.D.)**	**Mean**	**RW**	**Mean**	**RW**
Total		903 376							
Gender	Male	473 194	52.4	4.2	(3.7)	793.7	1.022	579.0	1.007
	Female	430 182	47.6	4.6	(3.6)	760.1	0.979	571.2	0.993
Age(in years)	<20	172 588	20.6	3.4	(2.2)	287.4	0.370	234.8	0.408
	21–30	147 523	14.9	3.4	(2.8)	350.1	0.451	287.9	0.501
	31–40	156 345	17.3	3.5	(3.0)	447.6	0.576	350.3	0.609
	41–50	155 422	17.2	3.9	(3.4)	642.6	0.827	468.7	0.815
	51–60	132 851	14.7	4.9	(3.9)	999.1	1.286	717.4	1.248
	61–70	68 509	7.6	6.6	(4.5)	1 670.6	2.151	1 177.2	2.047
	> = 71	70 138	7.7	8.4	(4.9)	2 613.7	3.365	1 894.6	3.295
Occupation	Employer	19 212	2.1	4.3	(3.6)	805.8	1.038	584.5	1.017
	Physician	2 531	0.3	3.6	(3.5)	802.0	1.033	556.2	0.967
	Paramedical personnel	15 826	1.8	4.0	(3.2)	576.6	0.742	439.4	0.764
	Public servant	48 080	5.3	4.2	(3.4)	708.6	0.912	517.3	0.900
	Employee or self-employed	667 980	73.9	4.1	(3.4)	657.8	0.847	489.3	0.851
	Farmer	92 441	10.2	5.9	(4.5)	1 262.8	1.626	917.5	1.596
	Fisherman	22 454	2.5	4.6	(3.7)	732.6	0.943	545.6	0.949
	Low income	34 852	3.9	6.5	(4.8)	1 960.8	2.525	1 466.1	2.550
Income	<=USD$594	226 634	25.1	4.3	(4.0)	916.2	1.180	679.4	1.182
	USD$594 - $ 1 210	464 094	51.4	4.5	(3.7)	762.3	0.982	566.5	0.985
	USD$1 210 - $3 850	203 846	22.6	4.1	(3.3)	658.4	0.848	482.6	0.839
	> = USD$3 850	8 802	1.0	3.7	(3.2)	676.1	0.871	476.8	0.829

Note: Distributions of Rx-MG counts and total cost were all statistically different among genders, age groups, type of occupation, and income class (p < .001); *RW* relative weight, the costs divided by the population mean.

Table 2 illustrates the total annual costs according to occupation, income, and multimorbidity status (based on data from 2010). According to the medication received in 2010, 114 442 of the enrolees (12.7%) received no Rx-MG assignment, while the remainder received at least one. Nearly 80% of the enrolees included in this data presented multimorbidity (i.e. had two or more Rx-MG assignments). Physicians and their unemployed family members had the lowest prevalence of multimorbidity (66.2%) and the highest prevalence of no Rx-MG assignment (22.9%). Low income earners had the highest prevalence of multimorbidity (86.8%). Enrolees in the first rank of income had the lowest prevalence of multimorbidity. As shown in Table 2, individuals with more Rx-MG assignments were associated with higher total healthcare costs. Similar to the results in Table 1, low income earners were associated with the highest total costs across all levels of multimorbidity, followed by physicians and farmers. Fishermans had the lowest mean total cost across all level of multimorbidity. Low income earners and farmers had the highest proportion (21.9% and 18.3%, respectively) of severe multimorbidity (i.e. > = 9 Rx-MGs). Individuals in the lowest rank of income had the highest total costs across all levels of multimorbidity. Similar results were obtained for each year from 2005 to 2009 (data not shown in Table 2).

**Table 2:** Annual total healthcare costs according to occupation, income, and multimorbidity status

	Annual total healthcare cost, by count of Rx-MGs^*^															
									**Multimorbidity**							
	**1Rx-MG**				**2-4 Rx-MGs**				**5-8 Rx-MGs**				**> = 9 Rx-MGs**			
	**n.**	**(%)**	**Mean**	**RW**^**a**^	**n.**	**(%)**	**Mean**	**RW**^**a**^	**n.**	**(%)**	**Mean**	**RW**^**a**^	**n.**	**(%)**	**Mean**	**RW**^**a**^
Total [n, (%)]	72 994	(8.1)	212.1	0.273	361 593	(40.0)	348.8	0.449	277 829	(30.8)	878.8	1.132	76 518	(8.5)	4023.8	5.181
Occupation																
Employer	1 663	(8.7)	204.7	0.264	7 359	(38.3)	363.7	0.468	6 175	(32.1)	978.0	1.259	1 602	(8.3)	3912.5	5.038
Physician	277	(10.9)	210.5	0.271	925	(36.5)	520.0	0.670	588	(23.2)	1233.5	1.588	162	(6.4)	4482.2	5.772
Paramedical personnel	1 568	(9.9)	198.0	0.255	6 765	(42.7)	341.4	0.440	4 759	(30.1)	819.0	1.055	766	(4.8)	3140.2	4.044
Public servant	4 314	(9.0)	197.5	0.254	20 646	(42.9)	357.0	0.460	14 979	(31.2)	836.4	1.077	3 353	(7.0)	3810.6	4.907
Employee or self-employed	56 375	(8.4)	202.7	0.261	276 311	(41.4)	329.8	0.425	199 394	(29.9)	814.2	1.048	43 970	(6.6)	3833.5	4.936
Farmer	5 288	(5.7)	225.3	0.290	29 972	(32.4)	377.1	0.486	32 366	(35.0)	1044.9	1.345	16 943	(18.3)	4121.1	5.307
Fisherman	1 604	(7.1)	191.0	0.246	9 203	(41.0)	313.2	0.403	7 382	(32.9)	764.6	0.985	2 073	(9.2)	3620.8	4.662
Low income	1 905	(5.5)	523.7	0.674	10 412	(29.9)	766.4	0.987	12 186	(35.0)	1571.7	2.024	7 649	(21.9)	5207.1	6.705
Income																
<=USD$594	17 679	(7.8)	267.2	0.344	82 347	(36.3)	425.1	0.547	64 322	(28.4)	1050.4	1.353	20 577	(9.1)	4755.8	6.124
USD$594 - $ 1 210	35 931	(7.7)	195.6	0.252	188 001	(40.5)	322.9	0.416	149 459	(32.2)	823.0	1.060	42 258	(9.1)	3780.2	4.868
USD$1 210 - $3 850	18 462	(9.1)	192.1	0.247	87 552	(43.0)	332.1	0.428	61 651	(30.2)	831.9	1.071	13 197	(6.5)	3654.7	4.706
> = USD$3 850	922	(10.5)	199.6	0.257	3 693	(42.0)	360.4	0.464	2 397	(27.2)	954.5	1.229	486	(5.5)	4232.8	5.450

*cost and count of Rx-MGs were based on the data of 2010.

^a^.RW: relative weight, the costs divided by the population mean.

Table 3 presents the results of mixed linear modeling intended to reveal the effects of multimorbidity and socioeconomic characteristics on log-transformed total annual costs. Model 1 controlled for age and gender, the results of which indicated that occupation and income level were associated with total healthcare costs, such that individuals with higher income incurred higher medical costs than those with lower income levels. Model 2, which also included multimorbidity status, identified a significant relationship between multimorbidity and total medical costs, as evidenced by a relationship between increases in the number of Rx-MG assignments and higher total costs. Compared to employees/self-employed enrolees, physicians, paramedical personnel, and public servants accounted for higher total costs after controlling for the multimorbidity and demographic characteristics, while employers, fishermen, and low income earners accounted for lower total costs. Unemployed individuals also accounted for higher total costs than employers and employees/self-employed enrolees. Model 3, which included the interaction between occupation and multimorbidity, indicated a moderating effect between the two variables, in which the higher income group was significantly associated with higher total costs. When only the influence of occupation was considered in the model (i.e. cases without multimorbidity and no moderating effect between the two variables), physicians, paramedical personnel, and public servants accounted for the highest total costs (all p < .001). Low income earners still accounted for lower costs than employees/self-employed (p < .001). The magnitude of parameters estimated in Model 2 and Model 3 demonstrate that multimorbidity accounted for the majority of variations in total healthcare costs.

**Table 3:** **Effects of multimorbidity and socioeconomic characteristics on annual total healthcare costs**^**a**^

	**Model 1**		**Model 2**		**Model 3**	
	**(β)**	**p-value**	**(β)**	**p-value**	**(β)**	**p-value**
Age	0.029	<.001	0.010	<.001	0.010	<.001
Male	−0.871	<.001	−0.353	<.001	−0.349	<.001
Female (reference)						
Occupation						
Employer	−0.096	<.001	−0.038	.006	0.768	<.001
Physician	−0.205	.007	0.582	<.001	2.692	<.001
Paramedical personnel	0.380	<.001	0.457	<.001	4.084	<.001
Public servant	0.364	<.001	0.151	<.001	3.027	<.001
Employees or self-employed (reference)						
Farmer	0.412	<.001	−0.025	.001	0.105	<.001
Fisherman	0.134	<.001	−0.104	<.001	0.042	.222
Low income	1.985	<.001	0.844	<.001	−0.377	<.001
Unemployed families (vs. employed)	0.876	<.001	0.302	<.001	0.304	<.001
Income						
<=USD$594	−1.191	<.001	−0.637	<.001	−0.622	<.001
USD$594 - $ 1 210 (reference)						
USD$1 210 - $3 850	0.206	<.001	0.221	<.001	0.219	<.001
> = USD$3 850	0.130	<.001	0.401	<.001	0.388	<.001
Multimorbidity						
0 RxMG (reference)						
1 - 4 RxMGs			11.650	<.001	11.884	<.001
5 - 8 RxMGs			12.685	<.001	12.917	<.001
> 8 RxMGs			13.798	<.001	14.010	<.001
Interaction of occupation and multimorbidity						
1 - 4 RxMGs * Employer					−0.916	<.001
1 - 4 RxMGs * Physician					−2.691	<.001
1 - 4 RxMGs * Paramedical personnel					−4.073	<.001
1 - 4 RxMGs * Public servant					−3.132	<.001
1 - 4 RxMGs * Farmer					−0.190	<.001
1 - 4 RxMGs * Fisherman					−0.155	<.001
1 - 4 RxMGs * Low income					1.192	<.001
5 - 8 RxMGs * Employer					−0.910	<.001
5 - 8 RxMGs * Physician					−2.564	<.001
5 - 8 RxMGs * Paramedical personnel					−4.167	<.001
5 - 8 RxMGs * Public servant					−3.252	<.001
5 - 8 RxMGs * Farmer					−0.143	<.001
5 - 8 RxMGs * Fisherman					−0.178	<.001
5 - 8 RxMGs * Low income					1.359	<.001
> 8 RxMGs * Employer					−0.992	<.001
> 8 RxMGs * Physician					−2.481	<.001
> 8 RxMGs * Paramedical personnel					−4.345	<.001
> 8 RxMGs * Public servant					−3.326	<.001
> 8 RxMGs * Farmer					−0.075	.001
> 8 RxMGs * Fisherman					−0.191	<.001
> 8 RxMGs * Low income					1.461	<.001
Model fitting indices						
−2 Log Likelihood	34 000 763		30 136 192		30 103 521	
AIC	34 000 805		30 136 234		30 103 563	
BIC	34 001 052		30 136 481		30 103 810	

^a^.costs are log-transformed.

Model 1: controlled for age and gender then estimated effect of occupation and income on log-transformed annual total healthcare costs.

Model 2: included all variables appeared in model 1, plus multimorbidity (Rx-MGs classification).

Model 3: included all variables appeared in model 2, plus the interaction between occupation and multimorbidity.

Table 3 outlines total annual costs and Table 4 summarizes the results of mixed linear modeling intended to reveal the effects of multimorbidity and socioeconomic characteristics on log-transformed non-pharmaceutical costs. After controlling for age and gender, Model 1 and Model 2 indicated that occupation, income level, and multimorbidity were all associated with non-pharmaceutical costs. Increases in the number of Rx-MG assignments and higher income were both associated with higher non-pharmaceutical costs. Similar to the results in Table 3, Model 3, which included the interaction between occupation and multimorbidity, indicated a moderating effect between the two variables. When only the influence of occupation was accounted for in the model, physicians, paramedical personnel, and public servants accounted for the highest total costs (all p < .001). Low income earners still accounted for lower costs than employees/self-employed (p < .001). Again, the magnitude of parameters estimated in Model 2 and Model 3 demonstrate that multimorbidity accounts for most of the variations in non-pharmaceutical costs.

**Table 4:** **Effects of multimorbidity and socioeconomic characteristics on annual non-pharmacy costs**^**a**^

	**Model 1**		**Model 2**		**Model 3**	
	**(β)**	**p-value**	**(β)**	**p-value**	**(β)**	**p-value**
Age	0.024	<.001	0.006	<.001	0.005	<.001
Male	−0.875	<.001	−0.362	<.001	−0.357	<.001
Female (reference)						
Occupation						
Employer	−0.099	<.001	−0.040	.003	0.781	<.001
Physician	−0.264	<.001	0.507	<.001	2.708	<.001
Paramedical personnel	0.366	<.001	0.439	<.001	4.046	<.001
Public servant	0.357	<.001	0.146	<.001	3.012	<.001
Employees or self-employed (reference)						
Farmer	0.389	<.001	−0.046	<.001	0.114	<.001
Fisherman	0.126	<.001	−0.111	<.001	0.030	.385
Low income	1.940	<.001	0.799	<.001	−0.350	<.001
Unemployed families (vs. employed)	0.833	<.001	0.250	<.001	0.251	<.001
Income						
<=USD$594	−1.193	<.001	−0.642	<.001	−0.627	<.001
USD$594 - $ 1 210 (reference)						
USD$1 210 - $3 850	0.209	<.001	0.224	<.001	0.222	<.001
> = USD$3 850	0.136	<.001	0.406	<.001	0.392	<.001
Multimorbidity						
0 RxMG (reference)						
1 - 4 RxMGs			11.503	<.001	11.741	<.001
5 - 8 RxMGs			12.503	<.001	12.742	<.001
> 8 RxMGs			13.646	<.001	13.854	<.001
Interaction of occupation and multimorbidity						
1 - 4 RxMGs * Employer					−0.925	<.001
1 - 4 RxMGs * Physician					−2.792	<.001
1 - 4 RxMGs * Paramedical personnel					−4.055	<.001
1 - 4 RxMGs * Public servant					−3.118	<.001
1 - 4 RxMGs * Farmer					−0.219	<.001
1 - 4 RxMGs * Fisherman					−0.151	<.001
1 - 4 RxMGs * Low income					1.111	<.001
5 - 8 RxMGs * Employer					−0.942	<.001
5 - 8 RxMGs * Physician					−2.717	<.001
5 - 8 RxMGs * Paramedical personnel					−4.138	<.001
5 - 8 RxMGs * Public servant					−3.246	<.001
5 - 8 RxMGs * Farmer					−0.192	<.001
5 - 8 RxMGs * Fisherman					−0.170	<.001
5 - 8 RxMGs * Low income					1.247	<.001
> 8 RxMGs * Employer					−1.019	<.001
> 8 RxMGs * Physician					−2.573	<.001
> 8 RxMGs * Paramedical personnel					−4.310	<.001
> 8 RxMGs * Public servant					−3.315	<.001
> 8 RxMGs * Farmer					−0.089	<.001
> 8 RxMGs * Fisherman					−0.183	<.001
> 8 RxMGs * Low income					1.422	<.001
Model fitting indices						
−2 Log Likelihood	33 906 909		30 075 330		30 042 645	
AIC	33 906 951		30 075 372		30 042 687	
BIC	33 907 198		30 075 619		30 042 935	

^a^.costs are log-transformed.

Model 1: controlled for age and gender then estimated effect of occupation and income on log-transformed annual non-pharmacy costs.

Model 2: included all variables appeared in model 1, plus multimorbidity (Rx-MGs classification).

Model 3: included all variables appeared in model 2, plus the interaction between occupation and multimorbidity.

## Discussion

This study explored the relationships among multimorbidity, socioeconomic status, and healthcare costs. The results of our mixed linear models with repeated measures clearly indicate that multimorbidity, occupation, and income were associated with variations in healthcare costs. A higher number of Rx-MG assignments was also associated with higher total costs. Occupation and income were associated with inequities in healthcare utilization.

Approximately 80% of the enrolees in this study had multimorbidity. The prevalence of multimorbidity in the literature varies widely between studies and much of this variance can be attributed to the method of data collection, operational definitions of multimorbidity, and the number of diseases/conditions selected [30-32]. Our findings are similar to those of Fortin et al. [33], in which the diseases adopted for the estimation of multimorbidity led to considerable discrepancies in the prevalence of multimorbidity, particularly in younger age groups. They found that when multimorbidity was measured using an open list of chronic diseases, instead of seven selected chronic conditions, the estimated multimorbidity prevalence increased dramatically from 17.1% to 73.9% for the 25–45 year age group. The Johns Hopkins ACG/Rx-MG incorporates all medications for the measurement of morbidity; however, the drugs used to treat acute diseases or symptoms (e.g. acute allergy or infection) were excluded from this study. Therefore, it is not surprising that the prevalence of multimorbidity estimated in this study was higher than that of other studies, which used lists of selected chronic conditions only.

This study employed pharmacy claims to obtain RxMGs assignment from the ACG system. Despite the wide-spread application of diagnosis-based measures of multimorbidity [4,34,35], these tools often suffer with regard to accuracy and quality in diagnosis coding [24,25,36,37]. Previous studies found that diagnoses identified using administrative data were highly specific but varied considerably in their sensitivity [37]. The ACG system also provides ADG assignments and has been used as a measure of multimorbidity [3,34], however it assigns the same ADG groups for different diseases, which is contrary to the theoretical underpinnings of multimorbidity, making it less meaningful within a clinical context. For example, ‘adult-onset type 1 diabetes’ (ICD9:250.00) and ‘essential hypertension’ (ICD9:401.9) are both assigned to ‘ADG10’ (chronic medical: stable), despite the fact that patients with only adult-onset type 1 diabetes or patients with the same disease as well as essential hypertension cannot be considered the same in terms of multimorbidity.

Our results indicate that socioeconomic status is associated with multimorbidity and healthcare costs. Farmers, fishermen, and low income earners had the highest proportion of moderate (5–8 Rx-MGs) to severe multimorbidity (> = 9 Rx-MGs). This result is consistent with those of other studies. van den Akker et al. found that low socioeconomic status, as measured by educational level, type of health insurance (as a proxy of income) and occupational status, is strongly associated with both morbidity and multimorbidity [38]. This study did not include educational level; however, it can be assumed that individuals working as physicians, paramedical personnel, and public servants are likely to be highly educated [2]. People with better education tend to be better informed with regard to health-related issues, which directly influences their lifestyle choices as well as the socioeconomic status of their families [39]. Testing of the Polychoric Correlation revealed that the correlation between occupation and income level is relatively low (−0.077). Our results also showed that income and occupation both have an independent impact on multimorbidity. This corresponds with the results of previous studies in which income was reported to be independently associated with health status after controlling for other socio-economic factors [11,40]. In this manner, multiple socioeconomic measures could be included simultaneously in multivariable models without (or with only marginal) problems of collinearity [40,41].

Our results also indicate that enrolees recognized by the government as low income earners had a higher prevalence of multimorbidity and increased healthcare costs when the RxMGs count increased. This adds to the evidence that lower income is associated with a higher prevalence of multimorbidity [8,11,12,31]. The greater utilization of healthcare among lower income groups may be due to the higher prevalence of multimorbidity.

This study also shows that after adjusting for age, gender, and multimorbidity, the interaction between occupation and multimorbidity revealed that low income earners had a positive moderating effect on the relationship between healthcare costs and increases in the count of RxMGs. We believe that this is because low income earners, who are eligible for a co-payment waiver, face a moral hazard with regard to the overuse of resources and tend to be isolated from the reality of increased healthcare costs. In this study, paramedical personnel, physicians and public servant accounted for higher medical costs. This study conducted sensitivity analysis to explore the odds of multimorbidity among the various occupations, stratified by calendar year. The results show that physicians and paramedical personnel faced lower odds of moderate and severe multimorbidity (> = 5 Rx-MGs). This may be explained by the fact that those with higher probability of higher education (e.g. public servant) or with more knowledge regarding the latest innovations in medicine (e.g. physicians and other medical professionals) are more likely to opt for more costly healthcare services and medications, such as brand-name drugs. Occupation, insurance status, and other individual-level socioeconomic characteristics are all closely linked to one’s access to, benefits from, and experiences with the health care system [42]. This study adds to a growing body of literature addressing inequities in the utilization of healthcare among patients from different socioeconomic backgrounds, regardless of their morbidity burden.

This study has several limitations. First, information related to the educational background of enrolees is unavailable in NHI claims data; therefore, the influence of this factor was not taken into account. Previous studies have indicated that educational differences can mitigate socioeconomic differences with regard to the utilization of healthcare. Although previous findings from studies on education and the prevalence of multimorbidity [2,11,12] have been inconsistent, future research on multimorbidity and socioeconomic status should nonetheless take this factor into account. Second, in this study, income was determined by monthly, salary-based figures. The Bureau of National Health Insurance has established a ceiling for the highest income group in the determination of premiums. As a result, the income values used to evaluate socioeconomic status may be biased, particularly for extremely high income earners and employees who receive bonuses as a major part of their income. Enrolees in different professions (e.g. blue-collar vs. white-collar) sharing the same employer will share the same code in the NHI data. This particular feature of the NHI claims database prohibited us from collapsing occupation categories into a more general form and may therefore limit the generalizability of the findings with regard to other countries. Finally, the high prevalence of multimorbidity found in this study may be due to the fact that the NHI pays for almost all prescription drugs and that the prescription data used in this study represents an aggregate from outpatient clinics, in-hospital pharmacies, and community pharmacies. The inclusion of such comprehensive data was intended to help capture all cases of acute and chronic morbidities. In addition, because medication costs represents a substantial portion (25%) of the total healthcare costs in Taiwan, the Rx-MGs assignments could effectively explain the variation in total healthcare costs. These unique features of Taiwanese NHI claims data might limit the generalizability of our findings.

## Conclusions

This study determined that multimorbidity and socioeconomic status are significantly associated with healthcare costs. In addition, an increase in the instances of multimorbidity (as measured by Rx-MG assignment) is associated with higher total healthcare costs. Inequities in healthcare utilization were observed between different occupations. Physicians, paramedical personnel, and public servant were found to utilize healthcare services to a greater degree than other groups. Low income earners accounted for significantly lower healthcare costs. The interaction between occupation and multimorbidity was also observed. Overall, these results highlight the fact that socioeconomic status is strongly associated with inequities in healthcare and should be considered in any discussion on the utilization of healthcare by patients with multimorbidity.

## Competing interests

Both authors declare that they have no competing interests.

## Authors’ contributions

KR contributed to the study design, statistical analysis, interpretation, and writing of the manuscript; LM contributed to the directing and coordination of the study, provided the algorithms for mapping NHI drug codes to WHO ATC codes, and interpretation and the writing of the manuscript. Both authors have read and approved the final manuscript.
